# Natural selection drove metabolic specialization of the chromatophore in *Paulinella chromatophora*

**DOI:** 10.1186/s12862-017-0947-6

**Published:** 2017-04-14

**Authors:** Cecilio Valadez-Cano, Roberto Olivares-Hernández, Osbaldo Resendis-Antonio, Alexander DeLuna, Luis Delaye

**Affiliations:** 1 Departamento de Ingeniería Genética, Centro de Investigación y de Estudios Avanzados del Instituto Politécnico Nacional, Unidad Irapuato, Km. 9.6 Libramiento Norte Carr. Irapuato-León, 36821 Guanajuato, Irapuato Mexico; 2 Departamento de Procesos y Tecnología, Universidad Autónoma Metropolitana-Cuajimalpa, Av. Vasco de Quiroga 4871, Santa Fe, Del. Cuajimalpa, C.P. 05348 Ciudad de Mexico, México Mexico; 3 Human Systems Biology Laboratory, Coordinación de la Investigación Científica-Red de Apoyo a la Investigación (RAI), UNAM, México City, Mexico; 4 Instituto Nacional de Medicina Genómica (INMEGEN), 14610 México City, Mexico; 5 Unidad de Genómica Avanzada (Langebio), Centro de Investigación y de Estudios Avanzados del IPN, Guanajuato, Irapuato Mexico

**Keywords:** Endosymbiont, Metabolic evolution, Adaptation, Metabolic integration

## Abstract

**Background:**

Genome degradation of host-restricted mutualistic endosymbionts has been attributed to inactivating mutations and genetic drift while genes coding for host-relevant functions are conserved by purifying selection. Unlike their free-living relatives, the metabolism of mutualistic endosymbionts and endosymbiont-originated organelles is specialized in the production of metabolites which are released to the host. This specialization suggests that natural selection crafted these metabolic adaptations. In this work, we analyzed the evolution of the metabolism of the chromatophore of *Paulinella chromatophora* by in silico modeling. We asked whether genome reduction is driven by metabolic engineering strategies resulted from the interaction with the host. As its widely known, the loss of enzyme coding genes leads to metabolic network restructuring sometimes improving the production rates. In this case, the production rate of reduced-carbon in the metabolism of the chromatophore.

**Results:**

We reconstructed the metabolic networks of the chromatophore of *P. chromatophora* CCAC 0185 and a close free-living relative, the cyanobacterium *Synechococcus sp*. WH 5701. We found that the evolution of free-living to host-restricted lifestyle rendered a fragile metabolic network where >80% of genes in the chromatophore are essential for metabolic functionality. Despite the lack of experimental information, the metabolic reconstruction of the chromatophore suggests that the host provides several metabolites to the endosymbiont. By using these metabolites as intracellular conditions, in silico simulations of genome evolution by gene lose recover with 77% accuracy the actual metabolic gene content of the chromatophore. Also, the metabolic model of the chromatophore allowed us to predict by flux balance analysis a maximum rate of reduced-carbon released by the endosymbiont to the host. By inspecting the central metabolism of the chromatophore and the free-living cyanobacteria we found that by improvements in the gluconeogenic pathway the metabolism of the endosymbiont uses more efficiently the carbon source for reduced-carbon production. In addition, our in silico simulations of the evolutionary process leading to the reduced metabolic network of the chromatophore showed that the predicted rate of released reduced-carbon is obtained in less than 5% of the times under a process guided by random gene deletion and genetic drift. We interpret previous findings as evidence that natural selection at holobiont level shaped the rate at which reduced-carbon is exported to the host. Finally, our model also predicts that the ABC phosphate transporter (pstSACB) which is conserved in the genome of the chromatophore of *P. chromatophora* strain CCAC 0185 is a necessary component to release reduced-carbon molecules to the host.

**Conclusion:**

Our evolutionary analysis suggests that in the case of *Paulinella chromatophora* natural selection at the holobiont level played a prominent role in shaping the metabolic specialization of the chromatophore. We propose that natural selection acted as a “metabolic engineer” by favoring metabolic restructurings that led to an increased release of reduced-carbon to the host.

**Electronic supplementary material:**

The online version of this article (doi:10.1186/s12862-017-0947-6) contains supplementary material, which is available to authorized users.

## Background

*Paulinella chromatophora* is an amoeba dispensed with phototrophic nutrition that contains blue-green photosynthetic organelles of cyanobacterial origin termed chromatophores [1, 2]. These novel organelles have a monophyletic origin in different strains of photosynthetic *Paulinella* that have been described [3] and were acquired through a primary endosymbiotic event about ~90 to 140 Mya [2–6].

Chromatophore genome sequencing from two strains of *P. chromatophora* (FK 01 [7] and CCAC 0185 [5]), revealed a size of 0.977 and 1.02 Mbp, respectively. This represents about 1/3 of the genome size of *Synechococcus sp*. WH 5701, the closest free-living relative cyanobacterium with a sequenced genome. *Synechococcus sp*. WH 5701 has a genome of ~3 Mbp and 3346 protein-coding genes [5]. It indicates that the chromatophore evolved by genome reduction. However, genome reduction in *P*. *chromatophora* is not as extreme as in plastids which rarely exceed 200 Kbp [2].

Chromatophores are genetically integrated with their host. More than 30 nuclear encoded genes of chromatophore origin have been identified [7, 8]. And some of the protein products coded by these genes are imported back into the chromatophore and participate in the photosynthetic apparatus [9]. Accordingly, chromatophores have been described as plastids in the making.

*P. chromatophora* nutrition relies on the reduced-carbon photosynthetically assimilated by the chromatophore [10]. This endosymbiotic-nutrient dependency has been observed in other organisms such as aphids and tsetse flies housing prokaryotic endosymbionts [11]. Particularly for aphids, host essential amino acids are provided by an endosymbiotic bacterium called *Buchnera aphidicola* [12]. Sequencing of the genome of *B. aphidicola* revealed a high degree of genetic degradation, while genes necessary for the syntrophic relationship with its host have been retained [12].

Prokaryotic endosymbionts evolve small genomes mainly by the combined action of genetic drift and negative selection [13–16]. In host-restricted conditions, the endosymbiont experiences a lack of recombination and horizontal gene transfer, as well as recurrent population bottlenecks lowering its effective population size (*N*_*e*_) and a concomitant relaxation of natural selection [15–17]. The combined action of these factors allows the accumulation of slightly deleterious mutations through a process called Muller’s ratchet [14, 17]. As a consequence, many genes become pseudogenes and are subsequently lost. In addition, selection at holobiont level by mechanisms like “partner fidelity feedback” have been proposed to promote the evolution of mutualistic interactions [18].

Something that should be considered is that, differing from free-living relatives, the metabolism of mutualistic endosymbionts is specialized in the production of metabolites that are released to their host as nutrients [19, 20]. This metabolic specialization is the consequence of metabolic restructuring caused by gene loss and genome reduction [20]. Resulting reduced genomes code for fewer genes, however, they are more integrated to the host. The extreme cases are organelles of endosymbiotic origin such as chloroplasts [21]. Therefore, if mutualistic endosymbionts show metabolic adaptations to provide nutrients to their hosts [19, 20], natural selection must have participated in the evolution of these systems.

During early stages of organellogenesis, the cyanobacteria that evolved into the chromatophore, had access to metabolites provided by the host. It is likely that the availability these metabolites render of some metabolic routes dispensable in the endosymbiont. The loss of these biosynthetic pathways in the endosymbiont led to restructurings and changes in the remaining metabolic fluxes. Taking into consideration all these modifications experienced by the chromatophore and the nutrient dependency of the holobiont for the photosynthetic function of the chromatophore, we made the analogy of natural selection acting as a “metabolic engineer” directing the strategies for the metabolic specialization of the chromatophore. In general, the objective of metabolic engineering is the directed improvement of metabolic capabilities through the deletion of metabolic genes or the introduction of new ones [22]. By using these strategies, microorganisms have been engineered for the improvement of the yield and the production and consumption rates of desired metabolites. For instance, for the of 1-butanol production in cyanobacteria [23], many more examples can be found elsewhere [24, 25].

In this work, we reconstructed the genome based metabolic models of the chromatophore of *Paulinella chromatophora* and the cyanobacteria *Synechococcus sp*. WH 5701. We inquired into the metabolic capabilities of the chromatophore; the possible metabolic interaction of the chromatophore with its host; and in silico simulate the process of metabolic evolution experienced by the chromatophore in host-restricted conditions.

## Results

### Differential gene retention of functional categories in the chromatophore genome

Our first objective was to determine to what extent genetic loss affected functional metabolic categories in the chromatophore (i.e. which functional gene categories were preferentially preserved) when compared to the genome of *Synechococcus sp*. WH 5701. We compared against *Synechococcus sp*. WH 5701 because is the closest free-living cyanobacterium with a sequenced genome and it is likely to be similar in gene content to the ancestor of the chromatophore. To assess the statistical significance we used a hypergeometric distribution.

**Fig. 1:**
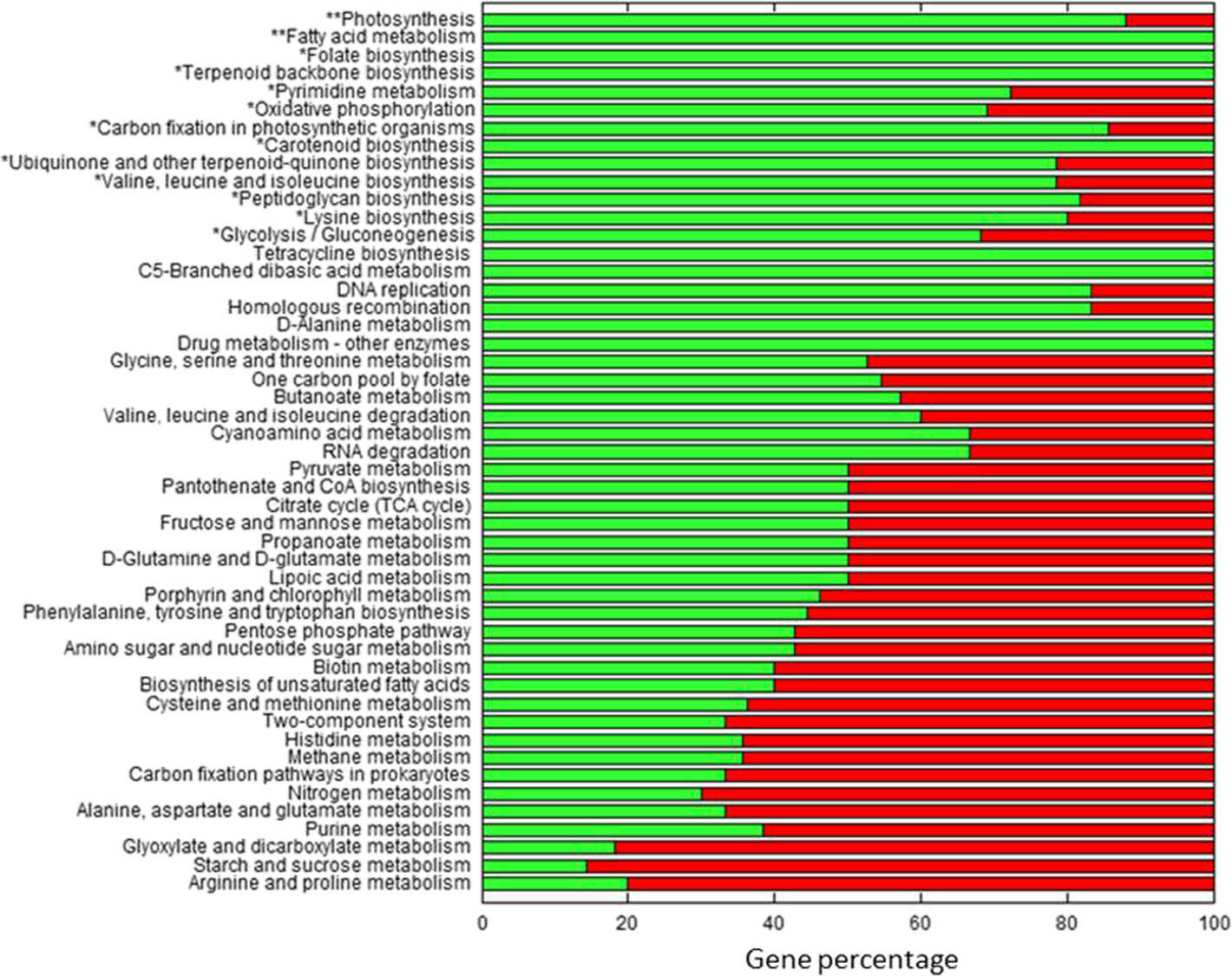
Conservation of functional gene categories in the chromatophore when compared to *Synechococcus sp*. WH 5701. For each functional category we show in green and red the percentage of gene conservation and lost in the chromatophore, respectively. For instance, if a gene category is completely green, it indicates that all orthologs in *Synechococcus sp*. WH 5701 are conserved in the chromatophore. As shown, gene loss affects differentially each one of the functional categories in the chromatophore. Functional categories particularly well conserved are indicated with asterisks (*p-value* < 0.05* or <0.05**, Bonferroni corrected). Statistical significance calculated by using a hypergeometric distribution [63]. Genes were classified following KEGG database (http://www.kegg.jp)

As is shown in Fig. 1, genes belonging to 13 functional categories have been less affected by genome erosion. In particular, photosynthesis and fatty acid biosynthesis categories are less affected. Retention of these 13 functional categories in the chromatophore can be attributed to a host-level selection protecting from gene loss. Conserved genes very likely play an adaptive role in the holobiont.

### In silico metabolic reconstruction of the chromatophore of *P. chromatophora* and *Synechococcus sp*. WH 5701

To better understand the role in the symbiosis played by remaining genes in the chromatophore, we reconstructed two metabolic models. One for the chromatophore of *P. chromatophora* CCAC 0185 [5] and the other for *Synechococcus sp*. WH 5701, the closest free-living cyanobacterium with a sequenced genome. The rationale behind this is to use *Synechococcus sp*. WH 5701 as a proxy of the ancestral cyanobacterium that evolved into the chromatophore.

Metabolic model reconstruction of the free-living cyanobacterium *Synechococcus sp*. WH 5701 was done by identifying orthologs to those protein-coding genes reported in the metabolic model of *Synechocystis sp*. PCC 6803 (*i*JN678) [26]. The resulting metabolic model of the free-living organism (*i*CV498) comprised 743 metabolic reactions with 698 metabolites and 498 protein-coding genes. Metabolic model reconstruction of the chromatophore was done by identifying those genes in the genome of the chromatophore of *P. chromatophora* CCAC 0185 that are orthologous to the free-living metabolic model (*i*CV498). The metabolic model of the chromatophore (*i*CV265) comprised 627 reactions, 615 metabolites and 265 protein-coding genes. Because *Synechococcus sp*. WH 5701 is a close free-living relative of the chromatophore, it could be considered that 158 reactions were lost along genome reduction in the chromatophore (Table 1).

**Table 1:** Characteristics of metabolic models of *Synechococcus sp*. WH 5701 (*i*CV498) and the chromatophore (*i*CV265)

	Metabolic model	
	*i*CV498	*i*CV265
Genes	498	265
Metabolites	698	615
Intracellular metabolites	661	578
Extracellular metabolites	37	37
Reactions	743	627
Enzymatic reactions	624	478
Transport reactions	82	70
Exchange reactions	37	37

By using the biomass equation of the cyanobacterium *Synechocystis sp*. PCC 6803 [26], we tested the functionality of the *i*CV498 and the *i*CV265 metabolic models with Flux Balance Analysis (FBA). Biomass production was set as objective function. In silico growth was simulated under autotrophic conditions. CO_2_ and photons uptake were set to 3.7 mmol × gDW^−1^ × h^−1^ and 100 mmol × gDW^−1^ × h^−1^ respectively and set as constraining metabolites as in [26].

In model *i*CV498, almost every metabolic pathway for biomass production is complete. The exceptions were 9 reactions for which no orthologous exist in *Synechococcus sp*. WH 5701 when compared to *i*JN678 (see model *i*CV498 in Additional file 1). These reactions had to be added to the *i*CV498 model in order to produce all the components necessary for the biomass equation. In this way, *i*CV498 showed an in silico growth rate of 0.0884 h^−1^ which is identical to the in silico growth reported for *Synechocystis sp*. PCC 6803 metabolic model under autotrophic conditions [26].

Under these conditions, the metabolic model of the chromatophore (*i*CV265) did not show in silico growth. This was obviously due to the reduced metabolic capabilities caused by the genomic reduction process experimented by the photosynthetic endosymbiont. Genome reduction has affected the metabolic capabilities of the chromatophore in two ways: a) some biosynthetic pathways were completely lost; while b) some other were partially lost.

**Fig. 2:**
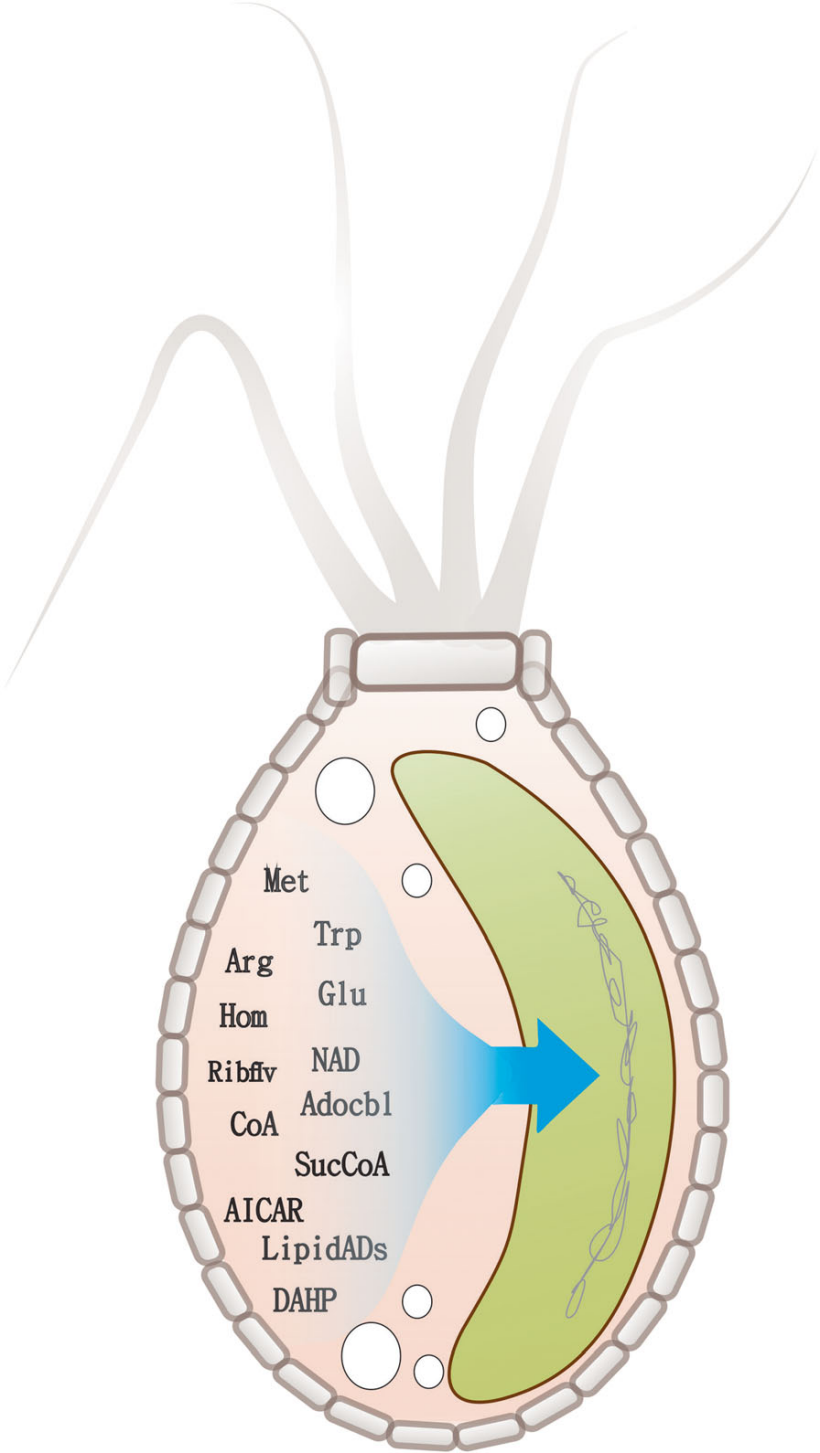
Nutrients uptake simulation in the chromatophore model (*i*CV265). Metabolites that cannot be produced by the chromatophore (with respect to the free-living model, *i*CV498) include: amino acids (Met = L-Methionine, Trp = L-Tryptophan, Arg = L-Arginine, Glu = L-Glutamate, Hom = L-Homoserine), cofactors (NAD = Nicotinamide adenine dinucleotide, Adocbl = Adenosylcobalamin, CoA = Coenzyme A), vitamins (Ribflv = Riboflavin) and others (AICAR =1-(5′-Phosphoribosyl)-5-amino-4-imidazolecarboxamide, SucCoA = Succinyl-CoA, LipidADs = Lipid A Disaccharide, DAHP =2-Dehydro-3-deoxy-D-arabino-heptonate 7-phosphate)

For example, in *Synechocystis sp*. PCC 6803 riboflavin is synthesized by four genes that perform six reactions by using Guanosine 5′-triphosphate (G5P) and D-Ribulose 5-phosphate (R5P) as precursors metabolites [26]. All these genes for riboflavin biosynthesis were lost in the chromatophore. In this case, we assumed that the host provides riboflavin to the chromatophore. The possible explanation for this loss is that riboflavin is the main precursor for flavin mononucleotide (riboflavin 5′-monophosphate, FMN) and flavin adenine dinucleotide, two main compounds that work as coenzymes for many of the enzymes such as oxidoreductases including NADH dehydrogenase as well as in biological blue-light photo receptors. This observation is concomitant with the loss in some functional gene categories; as in oxidative phosphorylation (Fig. 1). As the hypothesis is that the metabolic network must preserve its functionality, whenever we found a similar situation, exchange reactions were added to the metabolic model to simulate the incorporation of riboflavin and other metabolites as additional nutrients from the host. These metabolites included amino acids, cofactors, vitamins and other molecules which are essential for the biomass equation but cannot be produced by the chromatophore (Fig. 2).

Some other biosynthetic pathways are truncated in the chromatophore because single gene coding enzymes were lost. For example, in the biosynthetic pathway of leucine, most gene coding enzymes are present in the chromatophore except for the gene coding for 3-isopropylmalate dehydrogenase. In this case, we assumed that host encoded enzymes complement the pathway in the endosymbiont. Either by importing host encoded enzymes to the chromatophore or by exchanging intermediated metabolites between the symbionts. Similar situations have been proposed for other host-endosymbiont systems [12]. For this reason, we assumed that the production of these metabolites is shared between the host and the endosymbiont (see model *i*CV265 in Additional file 2).

In addition, some reactions in the chromatophore model *i*CV265 for which no orthologous genes exist with the free-living model *i*CV498 but are essential for in silico growth were assumed to be present (see model *i*CV265 in Additional file 2).

Finally, chromatophores lost the ability to store photosynthates as well as the capacity to synthesize sucrose [5]. Because of that, glycogen was removed from the biomass equation in *i*CV265. Under these conditions, in silico growth of the *i*CV265 model was 0.1568 h^−1^. This is an unrealistic rate because growth of the chromatophores is restricted to host division which is much lower than growth rate reported for free-living cyanobacteria and even other photosynthetic eukaryotes [27].

### Robustness analysis of metabolic models

**Fig. 3:**
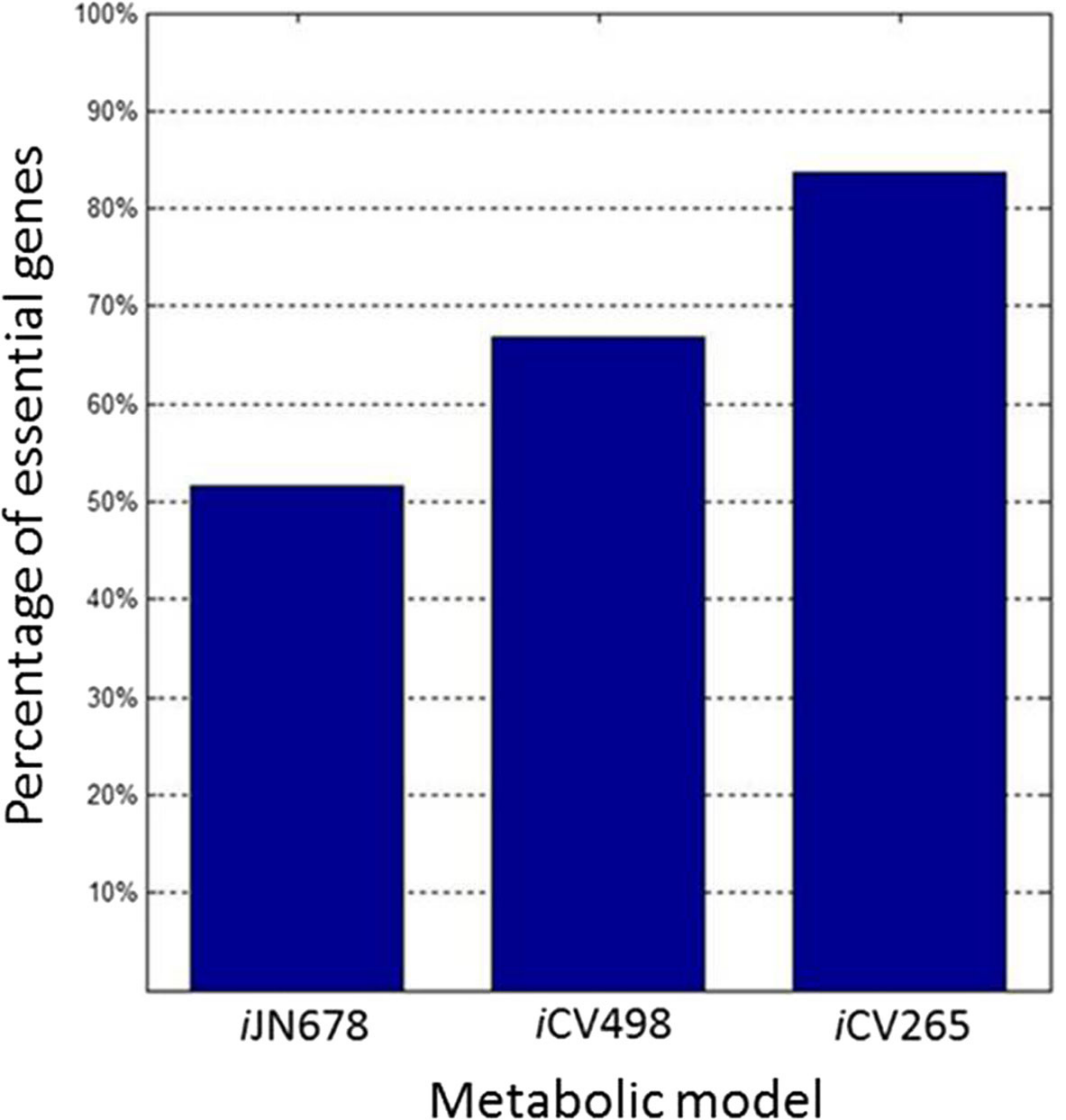
Genetic robustness analysis of metabolic models of *Synechocystis sp*. PCC 6803 (*i*JN678) [26], *Synechococcus sp*. WH 5701 (*i*CV498) and the chromatophore of *Paulinella chromatophora* (*i*CV265). Percentage of essential genes (Y axis) for each metabolic model (X axis)

We assessed the robustness of the *i*CV498 and the *i*CV265 models to single gene deletions. Genetic robustness was defined as the capacity of the models to maintain its metabolic capabilities (in silico biomass production) after a genetic deletion. Under phototrophic conditions, model *i*CV498 showed 333 genes (66.86%) to be essential because its deletion decreases the biomass production over 99% (Fig. 3). This result shows that *i*CV498 is less robust than the metabolic model of *Synechocystis sp*. PCC 6803 where 51.6% of the genes are essential under these same conditions [26]. In addition, there is a decreasing robustness in the model of the chromatophore where 222 of the 265 genes (83.77%) are essential (Fig. 3). This indicates that the genomic reduction experimented by the chromatophore rendered its metabolic network fragile. The same result has been observed for other metabolic networks of endosymbionts [20, 28, 29].

Interestingly, we found that there are 3 non-essential genes in the metabolic model *i*CV498 whose single deletion decreases in silico growth rate. These include genes encoding the enzymes acetyl-CoA synthetase, malic enzyme (NAD) and fumarase. Of these three, the last enzyme is the only one decreasing the in silico growth rate in *i*JN678 when it is deleted (data not shown). In the *i*CV265 model, all these 3 genes were lost. In addition, the non-essential gene in *i*CV498 coding for an enzyme with arginase activity is the only one whose deletion decreases the growth rate in the *i*CV265 chromatophore model. This suggests that genome reduction leading to *i*CV265 caused metabolic restructuring because deletion of this enzyme with arginase activity in *i*CV498 has no effect.

### In silico simulation of metabolic-gene loses in the chromatophore of *P. chromatophora*

Based on the metabolic network of the free living *Synechococcus sp*. WH 5701, we simulated in silico the gene loss. We evaluated the impact of intracellular conditions (metabolite availability) on the evolution of the chromatophore. In particular, we asked whether the set of metabolites predicted to be provided by the host in the *i*CV265 model (Fig. 2) determined actual gen content of the chromatophore after genome reduction.

Two in silico intracellular conditions were evaluated. In the first one, we simulated genetic reduction under in silico intracellular conditions where available nutrients were those predicted in the *i*CV265 model (we refer to them as Proposed Nutrients) (Fig. 2). In the second one, we randomly selected metabolites from the *i*CV498 model (the same number as in the first condition) and assigned as available nutrients under intracellular conditions (we refer to them as Randomized Nutrients; see Additional file 3: Table S1). The algorithm to simulate genome reduction is explained in detail in the methods section.

This algorithm allowed us to obtain in silico evolved chromatophores whose metabolic capabilities regarding the biomass production are equivalent to those of *i*CV265; but differing in their in silico evolutionary history and gene content.

Simulations under the Proposed Nutrients conditions resulted in reduced metabolic networks with 295.1 (± 2.63) genes on average. In these reduced networks, of the 498 genes present in the free-living ancestor (model *i*CV498), 52.2% are strictly conserved in the 500 simulations and 26.7% are dispensable in all of them. In Randomized Nutrients simulations, reduced networks have an average size of 326.8 (± 5.26) genes and 54% and 16.2% are conserved and dispensable in the 500 simulations, respectively.

**Fig. 4:**
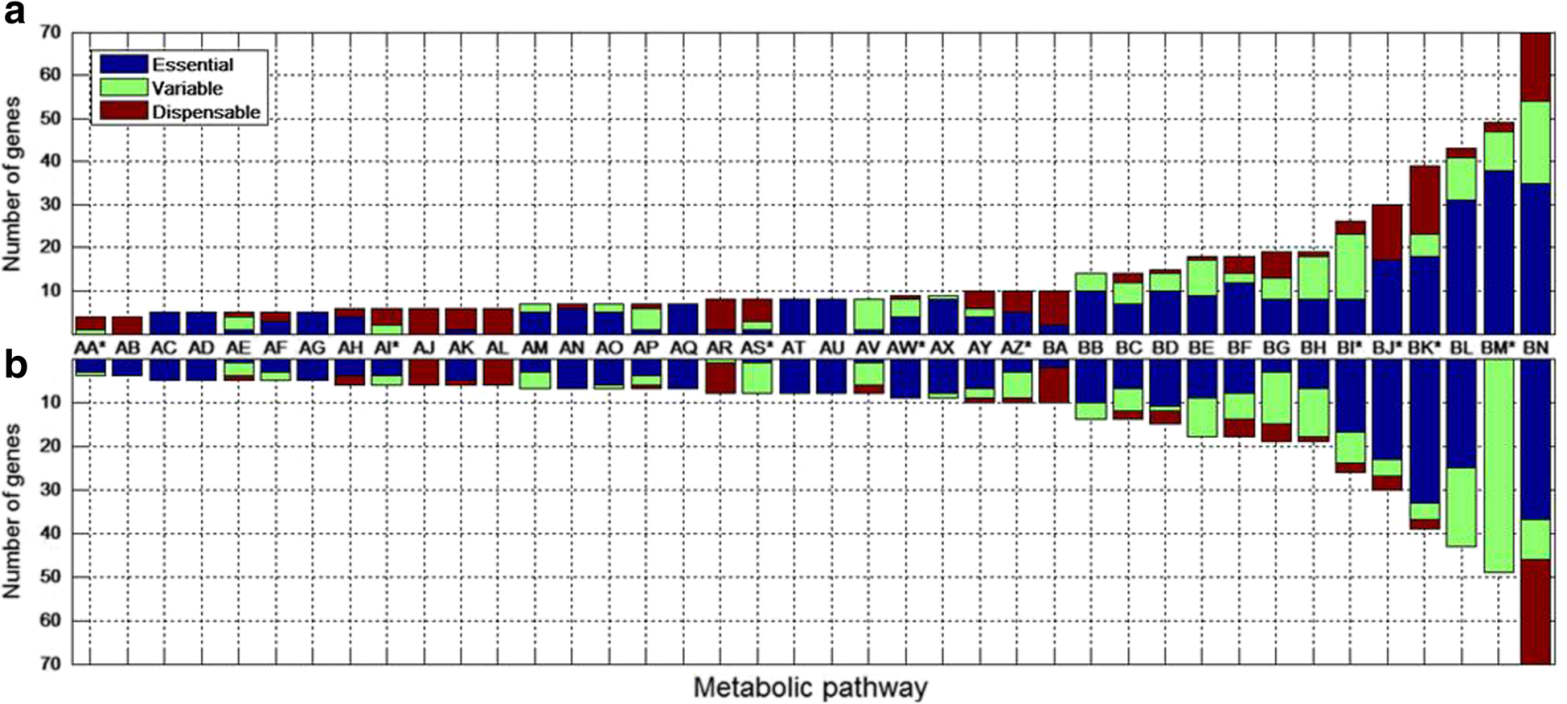
Variation in the proportion of genes classified as essential, variable and dispensable in different metabolic pathways according to two different sets of available nutrients for the chromatophore. **a** Set of Proposed Nutrients; **b** set of Randomized Nutrients. AA to BN metabolic pathways: AA* = Citrate cycle (TCA cycle); AB = Lipopolysaccharide biosynthesis; AC = Carotenoid Biosynthesis; AD = Folate biosynthesis; AE = Glycerolipid metabolism; AF = Hydrogen production; AG = Steroid biosynthesis; AH = Aminosugars metabolism; AI* = Nicotinate and nicotinamide metabolism; AJ = Nucleotide sugars metabolism; AK = Riboflavin metabolism; AL = Thiamine metabolism; AM = Carbon fixation; AN = Glutamate metabolism; AO = Lysine metabolism; AP = Nitrogen metabolism; AQ = Terpenoid backbone biosynthesis; AR = Fructose and mannose metabolism; AS* = Pantothenate and CoA biosynthesis; AT = Peptidoglycan biosynthesis; AU = Ubiquinone and other pterpenoids biosynthesis; AV = Urea cycle and metabolism of amino groups; AW* = Alanine, aspartate and glutamate metabolism; AX = Valine leucine and isoleucine biosynthesis; AY = Histidine metabolism; AZ* = Pentose phosphate pathway; BA = Starch and sucrose metabolism; BB = Fatty acid biosynthesis; BC = Glyoxylate and dicarboxylate metabolism; BD = Sulfur Cysteine and methionine metabolism; BE = Arginine and proline metabolism; BF = Pyrimidine metabolism; BG = Glycolysis/Gluconeogenesis; BH = Pyruvate metabolism; BI* = Phenylalanine tyrosine and tryptophan biosynthesis; BJ* = Purine metabolism; BK* = Porphyrin and chlorophyll metabolism; BL = Oxidative phosphorylation; BM* = Photosynthesis and BN = Transport. * Metabolic pathways with a difference in categorical genes with *p-value* < 0.05

As is shown in Fig. 4, the proportion of: i) essential genes (predicted to be essential in 500 simulations); ii) variable genes (predicted to be conserved in 1 to 499 simulations); and iii) dispensable genes (predicted to be lost in 500 simulations), varies between metabolic pathways. These proportions also vary between treatments (i.e.*,* Proposed Nutrients or Randomized Nutrients). Surprisingly, the most extreme case is that of the genes participating in photosynthetic activity. In Proposed Nutrients 77.6%, 18.3% and 4.1% are predicted to be essential, variable and dispensable, respectively. While for Randomized Nutrients none was predicted to be essential nor dispensable because 100% of them were variable.

**Fig. 5:**
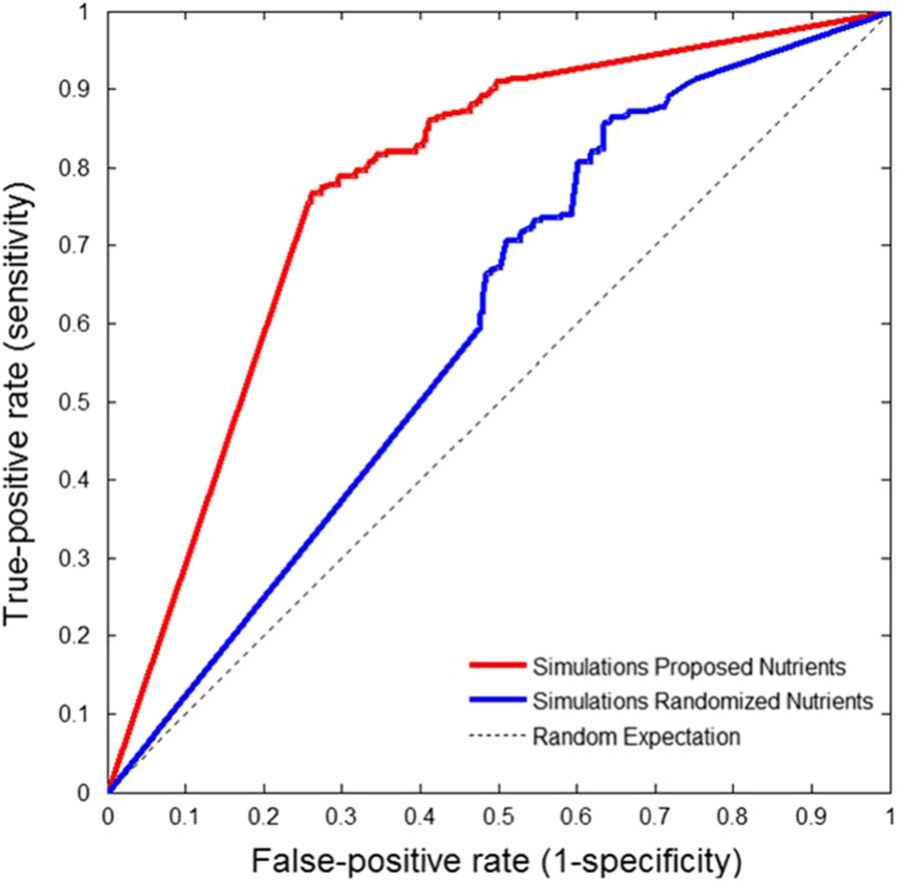
Genetic concordance between in silico evolved chromatophores and the chromatophore model (*i*CV265). The area under the curve indicates the predictive accuracy for evolved chromatophores (i.e. simulations) obtained under Proposed and Randomized Nutrients conditions. Proposed Nutrients: simulation with metabolites proposed as nutrients in the *i*CV265 model (Fig. 2) and Randomized Nutrients: simulations with randomized metabolites assigned as nutrients (Additional file 3: Table S1). Area under the curve: Proposed Nutrients =0.7742 (*p*-value = 2.853E-26); Randomized Nutrients =0.5987 (*p-value* = 1.42E-4)

Genetic concordance was evaluated between these simulated minimal networks and the *real* chromatophore model (*i*CV265). This was done by measuring sensitivity and specificity as in [30]. In Fig. 5, we show the fraction of true-positives and false-positives for every cutoff (1 to 500). True-positive and false-positive for every cutoff (1 to 500) form a curve whose area under the curve represents the probability that a gene conserved in *i*CV265 is present in more simulations than a gene which has been lost.

The area under the curve shows the contribution of the nutrients available in intracellular conditions explaining the evolutionary history experimented by the chromatophore. Accordingly, the accuracy obtained under the Proposed Nutrients condition was 77.4%, while that of the Randomized Nutrients was 59.8%. The difference between the areas under the curve from both conditions is statistically significant (*p*-value < 0.001, Chi-square test of homogeneity).

### Modeling selection and drift to explain metabolic evolution of the chromatophore

We are interested in understanding the role played by natural selection during the evolution of the metabolic capabilities of the chromatophore. Chromatophores provide the host with reduced-carbon, probably a hexose. This in analogy to the origin of plastids. It has been proposed that during the early stages of plastid evolution, the photosynthetic endosymbiont exported reduced-carbon in the form of an hexose-phosphate through an hexose phosphate transporter of bacterial origin (non-cyanobacterial) [31, 32].

To study how the potential rate of carbon exportation evolved, a hexose export reaction was added to the metabolic models. This reaction was defined as objective function. To ensure biomass components production, the biomass reaction was fixed to 0.0884 h^−1^ which, as stated previously, is the growth rate of *Synechocystis sp*. PCC 6803 metabolic model under autotrophic growth conditions [26].

**Fig. 6:**
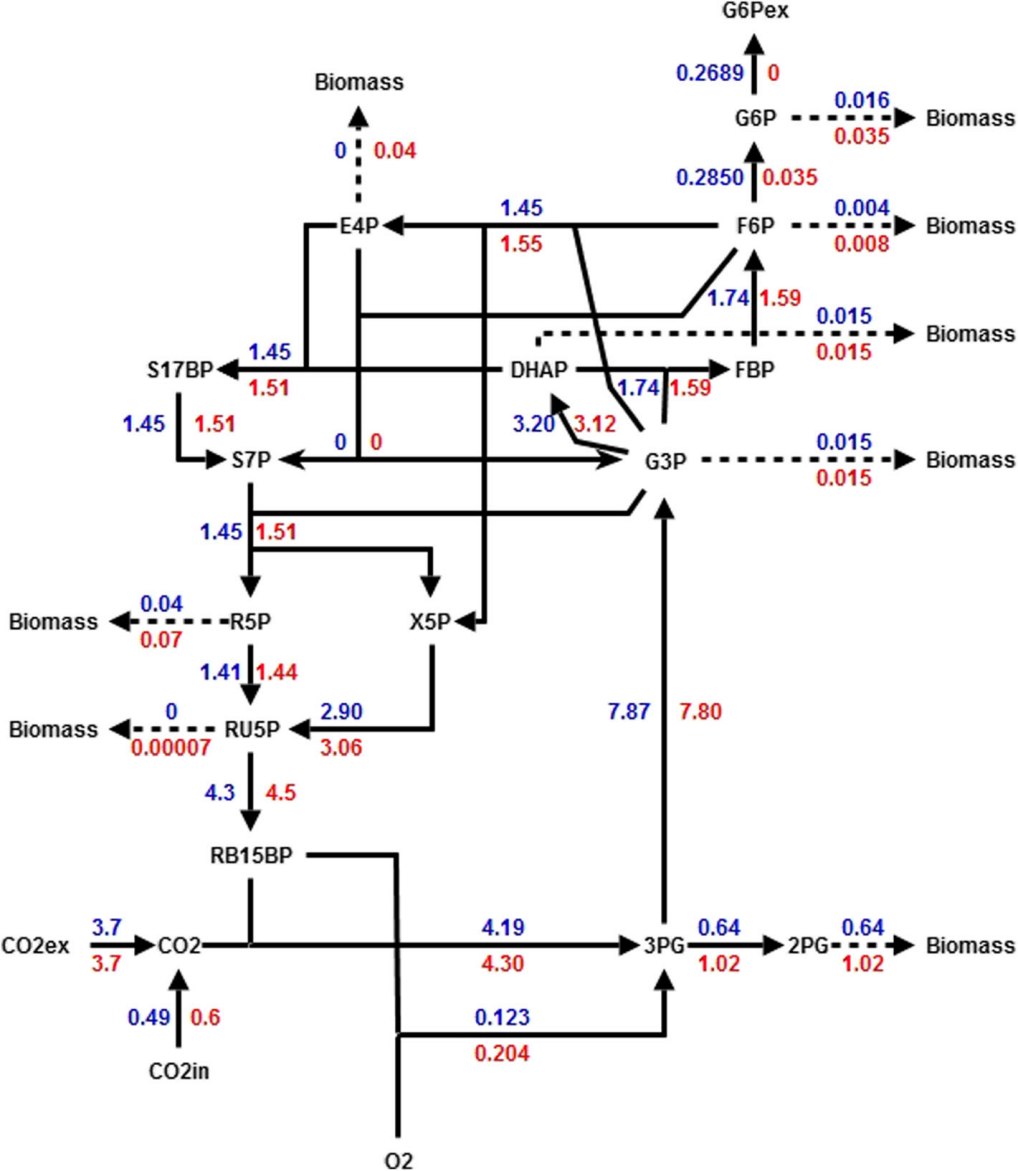
Flux distribution obtained with FBA of the central metabolism of *i*CV265 (*blue*) and *i*CV498 (*red*)

Under these conditions, there is no exportation of reduced-carbon in the *i*CV498 model. However, in the *i*CV265 chromatophore model, the potential rate of hexose exported without affecting the in silico growth rate was 0.2689 mmol × gDW^−1^ × h^−1^. In Fig. 6, we show the fluxes calculated with FBA of the central metabolism of the models of the chromatophore and the free-living cyanobacteria in conditions previously mentioned.

Fluxes calculated for production of metabolites precursors used to produce biomass components are produced in less quantity in the chromatophore’s model (Fig. 6). This is a consequence of the loss of metabolic capabilities in the metabolism of the chromatophore which allow the redirection of carbon through the gluconeogenic pathway for the production of hexoses as metabolic objective, instead of being used in the production of biomass components.

To analyze the efficiency of the metabolic networks in terms of hexose production at overall metabolism, we calculated the yields. The yields are parameters that measure the efficiency of the metabolic network and allow the comparison across different microorganisms. For instance, the yield of the ethanol production is higher in *Saccharomyces cerevisiae* compared to *Zymomonas mobilis*, this was the result of the specialization of the microorganism to produce specific metabolites [33]. Therefore, we calculated the yields of carbon, energy and reducing equivalents (extracellular CO_2_, ATP and NADPH) required to produce hexose (Table 2). These results show that the model of the chromatophore is more efficient for producing hexose from the external carbon than the free-living cyanobacteria. It means that metabolic restructurings experienced by the chromatophore rendered its metabolism more efficient to produce hexose which can be provided to the host.

**Table 2:** Yields (Y_P/S_) analysis of extracellular CO_2_, ATP and NADPH consumed in hexose production for both models

	Metabolic model	
	*i*CV265	*i*CV498
	mmol Hexose/ mmol	mmol Hexose/ mmol
CO_2_	0.069	0.009
ATP	0.023	0.002
NADPH	0.036	0.004

The yields suggest that the loss of some metabolic capabilities in the ancestral cyanobacterium caused a redirection of fixed CO_2_ causing changes in metabolic fluxes and consequently increasing the rate of reduced-carbon exported to the extracellular compartment.

We then inquired about the evolutionary forces that determined genetic conservation and metabolic functionality in chromatophores. Specifically, we wanted to infer if these metabolic capabilities of the *i*CV265 model (the potential rate of hexose exportation of 0.2689 mmol × gDW^−1^ × h^−1^) could have been possible under a random model of evolution or were the consequence of natural selection for metabolic specialization of the chromatophore and its positive impact at the holobiont. To tests this, we simulated the metabolic reduction process with hexose export and biomass production as evolutionary restrictions in a random model where hexose exported must be greater than zero. It means that every gene affecting the in silico growth rate of 0.0884 h^−1^ and impairing hexose export was considered as essential, while the hexose export rate could always vary while being greater than zero (purifying selection restriction for hexose export).

Minimal networks obtained in silico under these conditions were variable in size and gene content. Of 500 simulations, only 175 (66.03%) of the genes conserved in model *i*CV265 are conserved in all the simulations. Conversely, there are 45 genes predicted to be essential in all these 500 simulations which are not conserved in model *i*CV265. The metabolic networks from these 500 simulations are different in gene content and show different hexose export rates however they are equivalent in biomass production (Additional file 3: Figure S1).

**Fig. 7:**
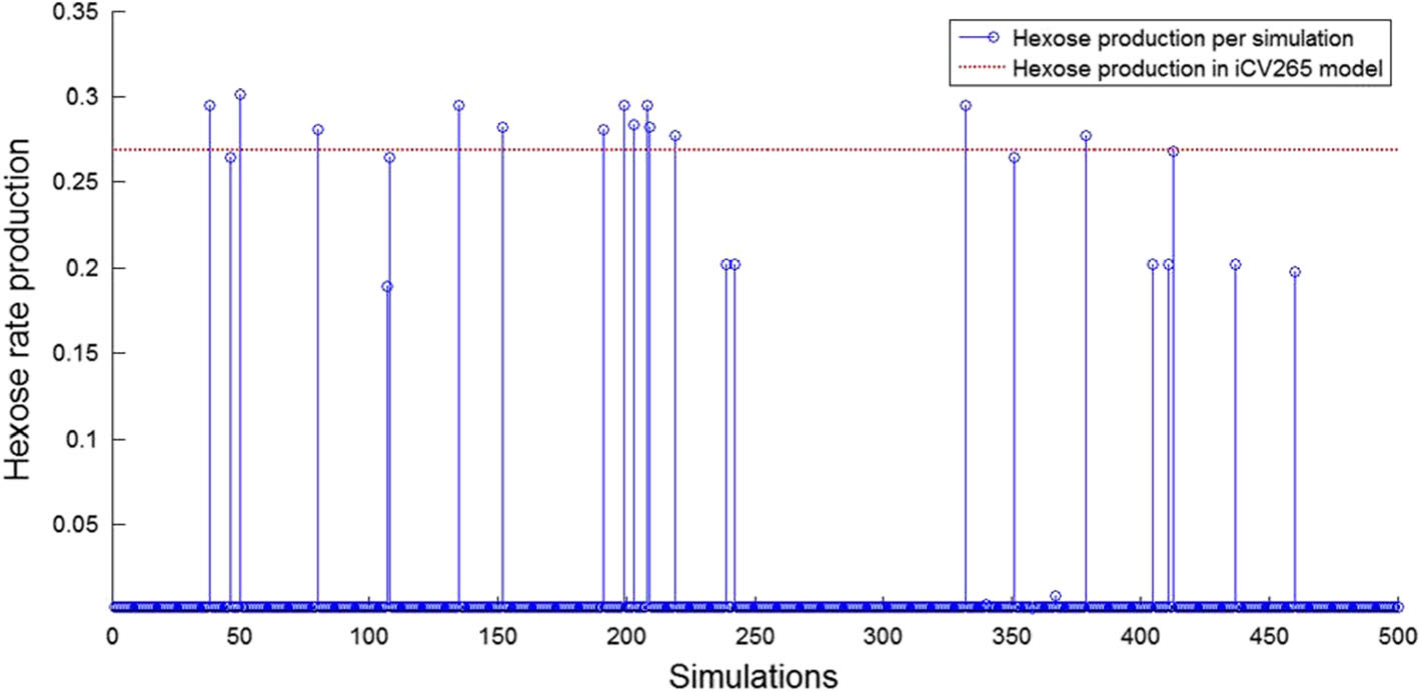
Hexose exportation rate of the chromatophore model (*i*CV265) is achieved in only 2.6% of the simulations under a random model of evolution. Hexose exported rate (Y axis) for 500 independent simulations (X axis). Red-dotted line indicates the hexose export rate in the chromatophore model (*i*CV265)

As shown in Fig. 7, after in silico metabolic reduction, hexose export rate in minimal networks obtained under these conditions tend to be minimal and close to zero (hexose export rate could not reach zero because of the restriction we imposed). On the other hand, only 2.6% of simulations have a potential rate of hexose exported equal or higher than the metabolic model of the chromatophore (0.2689 mmol × gDW^−1^ × h^−1^). This suggests that the probability of obtaining a potential rate of hexose exported similar to that of *i*CV265 under a random model is less than 5%. We got a similar result by varying the growth rate constraint of 0.0884 h^−1^ under plausible biological values (see Additional file 3: Figure S2).

Although variable, our simulations evolved metabolic networks that have approximately the same number of reactions than *i*CV265. The average number of reactions with non-zero fluxes in the reduced metabolic models of the 500 simulations is 416.15 ± 3.91. This is slightly less than the number of reactions with non-zero fluxes in the *i*CV265 model (442 reactions). This shows that the small percentage of simulations (2.6%) showing a potential rate of hexose exported equal or greater than that of the chromatophore (0.2689 mmol × gDW^−1^ × h^−1^) is not due simply to smaller size of the simulated metabolic networks.

These in silico experiments suggest that the potential rate of hexose exported in model *i*CV265 is unlikely to be the outcome of only genetic drift and purifying selection (i.e., less than 5% of the simulated networks export hexose at a rate comparable to that of *i*CV265). This suggests that the potential rate of hexose exported was the result of a process of functional specialization in which the increasing rate of hexose exportation was favored by natural selection due to the positive impact at the holobiont level.

Interestingly all these 2.6% of in silico evolved chromatophores have in common the conservation of a phosphate transporter via ABC system which is also present in the chromatophore model (*i*CV265). Conservation of this phosphate transporter allows the simulated network to get the phosphate necessary to be able to export fixed carbon. Without this transporter most of fixed carbon is oxidized in the pentose phosphate pathway releasing only a small amount to the extracellular compartment (data not shown).

### Metabolic integration of the chromatophore to its host

In our previous simulations, we assumed that nutrients (Fig. 2) were available simultaneously for the chromatophore since the beginning of the evolutionary process at the onset of the endosymbiosis. However, it is likely that this has not been the case and transporters for these nutrients were gained (or lost) sequentially. For instance, metabolic transport activity in the chromatophore is reduced due to loss of most transporters in comparison to free-living cyanobacteria [5]. And it has been reported that a large percentage of solute transporters in plastids from Plantae have host and bacterial (non-cyanobacterial) origin [31, 34].

Therefore, we simulated the evolutionary acquisition of transporters and its consequences in gene loss and the capability of the chromatophore to export fixed carbon to its host.

For every simulation, we used *i*CV498 as a free-living ancestor of the chromatophore under nutrient-rich conditions (Fig. 2). However, in this experiment, the model *i*CV498 did not have access to all nutrients since the beginning of the simulation. Instead, we randomly assigned a transport allowing the uptake of the respective nutrient. We then randomly deleted one gene at a time from *i*CV498. If the deleted gene affected the growth rate (0.0884 h^−1^) or impaired hexose exportation, we considered this gene as essential and we restored it to the model. In this way we analyzed the selective impact caused by gene loss due to the addition of a single transporter and the concomitant relaxation of natural selection for retention of specific biosynthetic pathways. Once we analyzed every gene in the model, we randomly assigned a second transport and then we repeated the gene loss simulation mentioned above. Simulation stops when in silico chromatophore has access to the 13 nutrients (Fig. 2) and all genes have been evaluated for their essentiality.

**Fig. 8:**
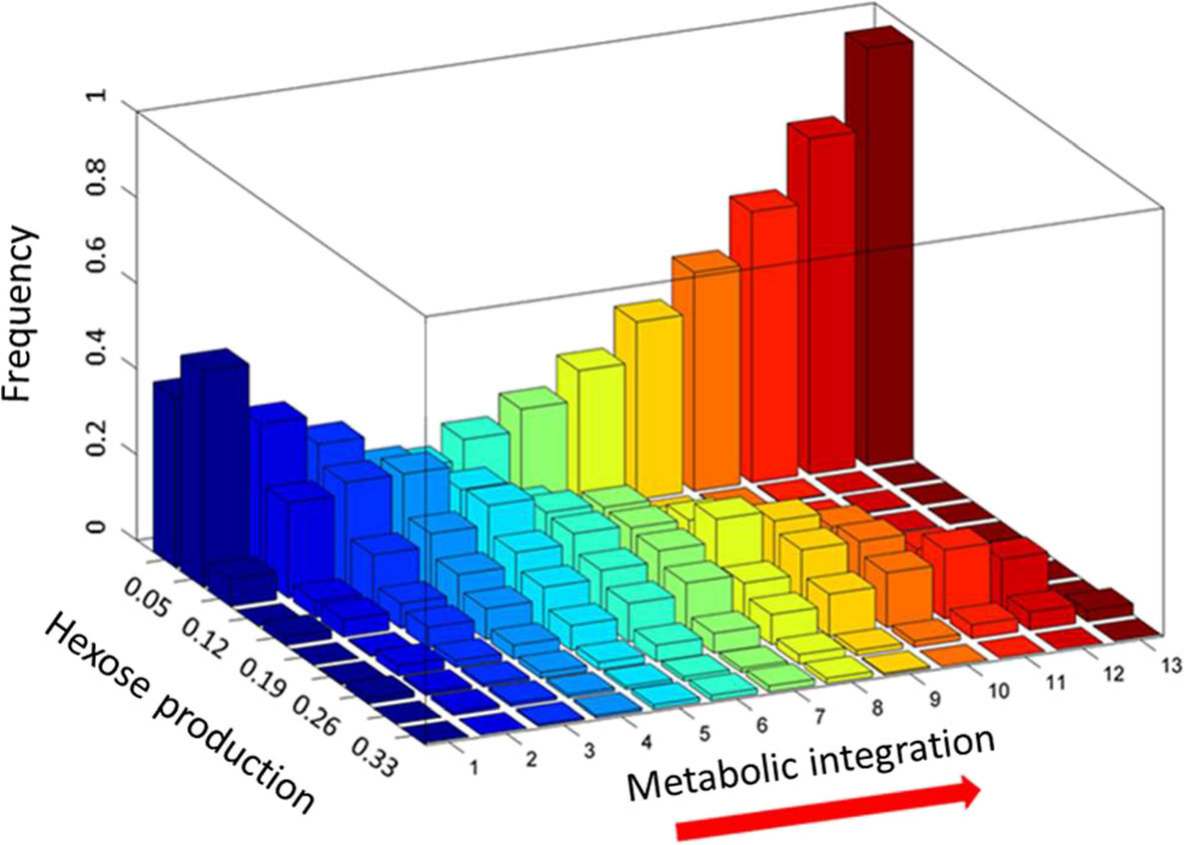
Metabolic integration with the host determines the rate of hexose exported by in silico chromatophores as well as the frequency of simulations that provide higher rates of reduced-carbon to the host. X axis, hexose export rate; Y axis, frequency of simulations; Z axis, transporters added to the model (metabolic integration)

As shown in Fig. 8, after the incorporation of the 13 transporters, the probability of getting a potential rate of hexose exported equal or higher than the metabolic model of the chromatophore (0.2689 mmol × gDW^−1^ × h^−1^) is less than 5% in 500 simulations, in agreement with our previous result (Fig. 8).

During the process of metabolic integration, it is noted that the maximum rate of hexose exportation becomes greater with every metabolite obtained as nutrient. However, by inspecting the frequency distribution of simulations with different potential rates of hexose exported, it is obvious that as metabolic integration advances, the probability of getting the maximum rate of hexose exportation decreases (i.e. the frequency of networks with large export rate becomes smaller).

These changes in frequency distributions during the process of metabolic integration can be interpreted in terms of the functional specialization of the chromatophore. As metabolic integration proceeds (with the addition of more transporters), the chromatophore increased its capacity to provide fixed carbon to its host. However, continued gene loss led to a simplified metabolic network and a smaller fraction of in silico evolved chromatophores can export as much fixed carbon as *i*CV265. The evolutionary landscape becomes smaller as evolution proceeds.

### The metabolism of the chromatophore is specially adapted to produce carbon for its host

As shown above, the potential rate of hexose exported in the chromatophore model (*i*CV265) is highly dependent on phosphate consumption. In addition, the growth rate of the chromatophore is coupled to the host’s growth rate. As shown above, the potential rate of hexose exported is unlikely to be the outcome of a random evolution.

**Fig. 9:**
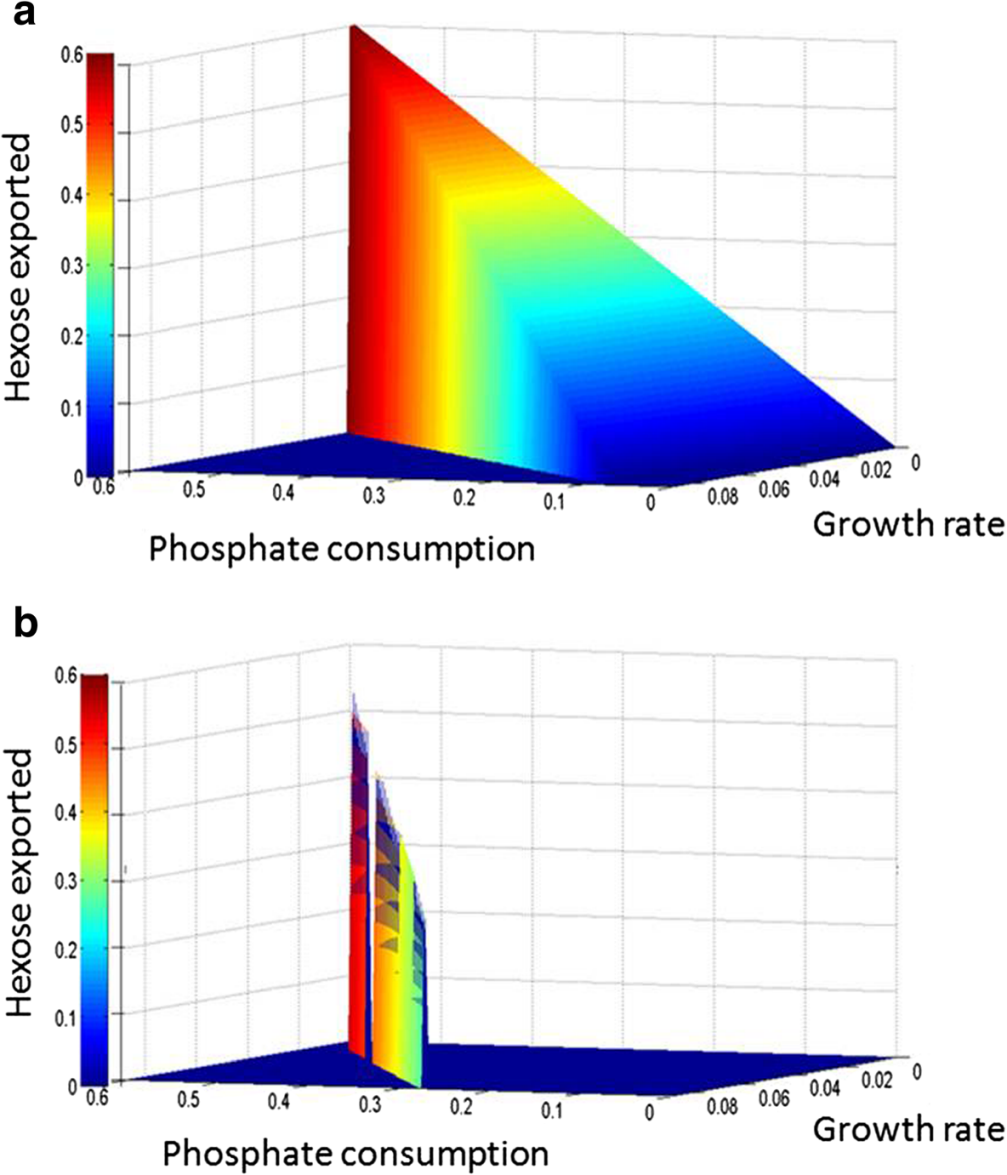
The metabolic model of the chromatophore (*i*CV265) is specially adapted to produce higher rates of reduced-carbon under phosphate and growth restrictions in comparison with *Synechococcus sp*. WH 5701 (*i*CV498). **a** Hexose export rate in the free-living model *i*CV498. **b** Hexose export rate in the chromatophore model *i*CV265. Y axis, growth rate restriction. X axis, phosphate uptake restriction. Z axis, potential rate of hexose exported. Colors in axis Z are only for drawing purposes and do not represent an extra variable

To test the impact of these two restrictions, we analyzed the metabolic properties of models *i*CV265 and *i*CV498 in potential rate of hexose exported under growth rate and phosphate uptake restrictions. As shown in Fig. 9, the potential rate of hexose exported in the *i*CV498 model is robust with respect to growth rate and phosphate uptake (i.e. a given growth rate can sustain the hexose rate exportation with different rates of phosphate consumption). This contrasts with the chromatophore model (*i*CV265) where, for a given growth rate, only a specific consumption of phosphate is necessary to sustain hexose release. In addition, the capacity of hexose export in the *i*CV265 model for a determined growth rate restriction is greater than the *i*CV498 model.

## Discussion

In this work, we show that the metabolic network of the chromatophore of *P. chromatophora* is different to the metabolic network of its free-living relative *Synechococcus sp*. WH 5701. We suggest that these differences evolved by natural selection. Gene-loss and carbon flux redirection guided by natural selection led to metabolic specialization of the chromatophore as a reduced-carbon provider.

### Purifying selection and the maintenance of the symbiosis

Our analysis showed that some metabolic pathways have been preferentially conserved in the chromatophore (Fig. 1). These preserved metabolic pathways (i.e. photosynthesis, carbon fixation, and gluconeogenesis) very likely play a prominent role in the symbiosis. This pattern is analogous to the one observed in many other endosymbionts e.g. *Buchnera aphidicola* [35]. In this later case, biosynthetic pathways producing essential amino acids for the host [12, 35] are preserved by host-level natural selection.

As mentioned above, differential conservation of gene category functions suggests that purifying selection is preserving relevant symbiotic functions. Accordingly, estimation of the rate of nucleotide substitution in 681 DNA alignments of protein-coding genes orthologous between chromatophores of two different strains of *P. chromatophora* (CCAC 0185 [5] and FK 01 [7]) showed that most of them have signals of purifying selection [7].

It has been suggested that host-level selection prevents the fixation of deleterious mutations in endosymbionts thus lowering the chances of a mutational meltdown resulting in extinction [36, 37]. And, of course, this prevents the consequent replacement of non-functional endosymbionts [38]. In addition, selective pressure to maintain functional proteins increases with the time of host-endosymbiont interaction [36] and combined with very strong bottlenecks may help to reduce the accumulation of deleterious mutations. This has been proposed to explain mitochondrial genome evolution [39].

### Metabolic integration of the chromatophore to its host

Comparison of the metabolic models of the chromatophore and the cyanobacterium *Synechococcus sp*. WH 5701 allowed us to inquire into the evolution of the metabolic interaction of the chromatophores with its host. For example, several metabolic pathways in the chromatophore are incomplete. It is likely that the host supplies these metabolites as nutrients to the chromatophore. Metabolic pathway sharing is a hallmark of endosymbiotic organisms. For example *Wolbachia*, which are endosymbionts of many animal species, show a degraded genome [40, 41] whose limited metabolic capabilities are complemented by its host [42]. In turn, the endosymbiont provides the host with nutrients such as riboflavin, positively impacting host fitness [42]. Equally remarkable is the likely coupled production of some metabolites between the chromatophore and its host. As mentioned above, this collaboration in metabolite biosynthesis has been observed in other symbiotic systems [43–46].

### Fragility of a reduced metabolic network

To study the metabolic capabilities of the chromatophores we used FBA. This stoichiometric approach can predict cellular phenotypes in specific environmental conditions. Generally, biomass production is fixed as objective function. In absence of biomass composition, the use of a biomass equation from a related organism is a valid starting point for metabolic analysis [47–49]. In this way, FBA has been used to infer the metabolic capabilities of different organisms whose cultivation and experimental management is challenging or not yet possible, as in the case of endosymbionts. For example, biomass composition and the metabolic model of *Escherichia coli* were used for metabolic analysis of *Buchnera aphidicola* [20, 30], *Sodalis glossinidius* [29], and *Blattabacterium cuenoti* [28]. In the same way, we used the biomass composition and stoichiometric model of *Synechocystis sp*. PCC 6803 as a starting point to model the metabolism of the chromatophore and *Synechococcus sp*. WH 5701 [26].

We found that the metabolism of the chromatophore is highly fragile to gene deletions. Approximately 84% of the genes in the model are essential when singly deleted in comparison with ~67% of the genes in *Synechococcus sp*. WH 5701. A similar difference in metabolic fragility was found by [20] when comparing the models of *B. aphidicola* and its free-living relative *Escherichia coli* where 84% and 19% of genes were essential, respectively. In the same way, the metabolic network of two strains of *Blattabacterium cuenoti* (Bge and Pam), the obligated primary endosymbiont of cockroaches, were shown to be highly fragile to single gene deletion. It was found that 76.1% and 79.6% were essential genes, respectively [28]. Finally, in *Sodalis glossinidius* (the secondary non-obligated endosymbiont in early stages of tsetse flies), 44.54% metabolic genes were found to be essential, compared with its ancestral network where only 25.48% are predicted to be essential [29].

Our robustness analyses of the *i*CV265 and the *i*CV498 models agree with the generalization that metabolic networks of endosymbionts are more fragile than their free-living counterparts. This metabolic fragility of endosymbionts contrasts with theoretical estimations that suggest that, in general, metabolic systems are robust and complex [50]. However, the metabolic systems of endosymbionts are considered more robust [28] than minimalist metabolic networks [51]. The difference in metabolic fragility of the chromatophore when compared to *Synechococcus sp*. WH 5701 reflects the transition from a free-living style to a more stable condition inside *Paulinella chromatophora*.

### Metabolic environment as a determinant of gene content

It has been shown that retention of metabolic genes in endosymbionts is determined by the metabolic requirements and molecular environment of the host [52, 53]. With the use of FBA and the metabolic model of *Synechococcus sp*. WH 5701 as a proxy of the ancestor of the chromatophore, we evaluated the impact of the host-metabolic environment in the reduction of the metabolic system of the endosymbiont. The proposed host-metabolic environment (Proposed Nutrients) predicted with 77.42% of accuracy the actual gene content of the chromatophore. This is in contrast with the 59.8% of accuracy obtained when using a randomly set of host-provided metabolites (Randomized Nutrients). This emphasizes the contribution of the intracellular metabolic environment to the evolution of the metabolism in the chromatophore.

Similar reductive simulations have been used to predict the set of essential genes of pathogens located in certain environmental niches (like the bloodstream) within the human body [52]. In the same way, reductive evolution simulations using *E. coli* as free-living ancestor predicts with 80% of accuracy the metabolic gene content of *B. aphidicola* and *Wigglesworthia glossindia* [30].

Inspection of the proportion of dispensable, variable, and essential genes by in silico reductive simulations (i.e. Proposed Nutrients and Randomized Nutrients) predicts differential gene retention patterns between different metabolic pathways. For example, in Randomized Nutrients simulations, photosynthesis pathway (which is the *raison d’être* of the symbiosis) 100% of genes are predicted as “variable” (none of the genes are predicted to be retained in the 500 simulations) while in Proposed Nutrients ~78% are essential. This means that under Randomized Nutrients, photosynthesis function could be useful but not essential and could have been lost in the chromatophore by chance. Clearly, the set of metabolites comprising Randomized Nutrients cannot account for the metabolic gene content of extant chromatophores.

Maximization of biomass production is regularly used as objective function in FBA analysis. It allows predicting the distribution of fluxes through a metabolic network [54]. The maximization of biomass function is used as a proxy of evolutionary fitness. However, many other objective functions can be used [54, 55]. For instance, it was estimated that *Chlorella* (the photosynthetic endosymbiont of *Paramecium bursaria*), releases 57% of its photosynthates to its host [56]. This means that most carbon photosynthetically assimilated is destined to symbiotic interaction instead of biomass production of *Chlorella* itself. In the same way, *P. chromatophora* has phototrophic nutrition. It depends on carbon assimilates which derivate from the endosymbiotic cyanobacterium whose inorganic carbon rate assimilation is the same as a free-living cyanobacteria [10]. But unlike its free-living relatives, its growth rate is restricted by *P. chromatophora*. Considering the above metabolic analysis of the chromatophore, which predict an in silico growth rate of 0.1568 h^−1^, it is difficult to consider the biomass as the only objective function in chromatophores. Taking into consideration that chromatophores provide the host with reduced-carbon, a reaction simulating hexose export to extracellular compartment was added. This reaction was defined as objective function. And to ensure biomass components production, biomass reaction was fixed to 0.0884 h^−1^ which is the growth rate of a free-living relative cyanobacterium. Interestingly, under these conditions the metabolic model of the chromatophore predicts a potential rate of hexose exportation of 0.2689 mmol × gDW^−1^ × h^−1^. As far as we know, this is the first metabolic reductive evolutionary analysis where metabolic functionality (i.e. hexose export) of the endosymbiont is explored as objective function, differing from previous analyses where biomass is set as objective function of mutual endosymbionts as *B. aphidicola* [20, 30], *S. glossinidius* [29] and *B. cuenoti* [28].

### ABC phosphate transporter is an essential component of the chromatophore

All simulations showing a hexose exportation rate equivalent to that of the chromatophore model (*i*CV265) share the ABC phosphate transporter. This P_i_-dependency in the chromatophore agrees with that observed in isolated spinach chloroplasts [57]. It has been shown that photosynthesis declines dramatically (less than 10% of the maximum rate) in chloroplast in the absence of P_i_ in the reaction medium. Also, carbon export from the chloroplast is inhibited [58], with up to 60% of ^14^C fixed being retained in the chloroplast [57]. As mentioned above, this observation agrees with the more than 95% of simulations which predict that lack of ABC phosphate transport favors carbon retention in the chromatophore instead of being released to the host. Therefore, we predict that lack of ABC transporter in the genome of the chromatophore of *Paulinella* FK01 is compensated by a phosphate transporter coded in the host [7].

### The role of natural selection on the evolution of the metabolism of the chromatophore

Inspection of FBA calculated central metabolic fluxes in the chromatophore and in the free-living cyanobacteria showed that the endosymbiont is better at producing hexose. This is likely a host related adaptation. To investigate whether this and other characteristics of the metabolic model of the chromatophore evolved by natural selection, we simulated in silico reductive evolution with a null model not including positive selection. As a proxy of genome reduction by purifying selection and random genetic drift, we submitted the metabolic model of *Synechococcus sp*. WH 5701 with the following algorithm: a) first, we simulated host-level purifying selection by requiring that the rate of hexose exportation of the model must be always greater than 0 and biomass is produced at 0.0884 mmol × gDW^−1^ × h^−1^; b) next, we performed rounds of single gene deletion until no more genes could be deleted; c) finally, we repeated this process 500 times. By this, we obtained a population of 500 reduced metabolic networks all of them capable of producing 0.0884 mmol × gDW^−1^ × h^−1^ of biomass, but differing in hexose rate exportation. Differences in rates of hexose exportation were due to contingency-dependent loss of alternative pathways [30]. With this experiment, we could determine if the potential rate of hexose exported in *i*CV265 (0.2689 mmol × gDW^−1^ × h^−1^) is easily obtained by host-level purifying selection (hexose exportation >0) and contingency-dependent evolution on random gene deletion. Our evolutionary reductive analyses showed that <5% of simulations were predicted to export hexose at a similar rate as the model *i*CV265. This suggests that metabolic functionality of *i*CV265 is unlikely to be determined by genetic drift alone. Therefore, we conclude that natural selection at holobiont level may have contributed to shape metabolic functionality of the chromatophore.

### Natural selection as metabolic engineer

According to the above mentioned, we consider suitable to make the analogy of natural selection as metabolic engineer. Metabolic engineering can be defined as “the directed improvement of product formation or cellular properties through the modification of specific biochemical reactions or introduction of new ones” [22]. One of the objectives of metabolic engineers is to redirect the flux of mass through the metabolism of organisms towards a desired metabolic product. Some genetic strategies to redirect metabolic flux toward production of a desired metabolite include: increasing the precursor supply; altering the regulation (overexpressing) genes; increasing the efficiency of bottleneck enzymes; reducing flux toward unwanted byproducts; or eliminating competing pathways by gene-deletion [59]. It has been proposed that cellular metabolism of free-living microorganisms is primed, through natural selection, for the maximum responsiveness to the history of selective pressures rather than for the overproduction of specific chemical compound [60]. In host-restricted conditions this responsiveness to free-living selective pressures are no longer needed. Instead, new biological objectives are defined now related to holobiont survival.

For instance, it was proposed that the chloroplast metabolic network has improved photosynthetic properties in comparison to free-living cyanobacteria [21]. For example, the metabolic network in chloroplast has: i) a longer average path length; ii) a larger diameter; iii) is Calvin Cycle-centered; iv) and presents better modular organization when compared with the network of free-living cyanobacteria [21]. In a similar way, the metabolism of the chromatophore (*i*CV265) seems to be tailored for the exportation of reduced-carbon; that is, when comparing the export of reduced-carbon between the *i*CV265 and the *i*CV498 models (with phosphate as restrictive nutrient) we found that *i*CV265 shows higher rates of hexose exported than the free-living *i*CV498 model at the cost of increased consumption of phosphate (Fig. 9).

The evolutionary mechanism outlined above applies when the host benefits from the endosymbiont. In particular, mechanisms such as “partner fidelity feedback” (PFF) promote cooperation between symbionts. PFF requires individuals to be “associated for an extended series of exchanges that last long enough that a feedback operates” [18]. Similar mechanisms likely operated in other symbiotic systems. For example, *Buchnera* [61] and *Blochmannia* [62] overproduce essential amino acids (EAAs) to its host. This overproduction of EAAs was consequence of metabolic restructurings due to metabolic-gene losses. For example, the truncation of the purine biosynthesis pathway which allows the endosymbiont to produce histidine at higher rates than free-living relatives [20]. Reductive evolutionary simulations carried out by [20] showed that this truncation is an improbable evolutionary event under conditions tested.

## Conclusion

Our main objective was to better understand the metabolic changes experienced by the free-living cyanobacteria to become a chromatophore. In addition, we assessed the evolutionary forces driving organellogenesis. We found evidence that certain metabolic pathways are preferentially conserved in the chromatophore. We also found that the pattern of metabolic gene loss strongly depends on the availability of nutrients from its host. The high fragility of the chromatophore network reflects the transition to a more stable environment and, consequently, its simplification. The chromatophore is specialized in producing reduced-carbon which could be released to the host. This specialization was consequence of metabolic restructurings which could not be possible in free-living conditions. We interpret this specialization as consequence of natural selection acting as a metabolic engineer which modifies intrinsic metabolic properties of the endosymbiont impacting positively at the holobiont level. Our in silico simulations allowed us to determine that metabolic specialization of the chromatophore is an unlikely result of purifying host-level selection and genetic drift alone. In this way, computational analysis of biological systems allows to obtain new insights on the evolutionary forces shaping metabolic evolution of mutualistic endosymbionts.

## Methods

### Differential gene retention of functional categories in the chromatophore genome

To identify metabolic pathways preferentially conserved in the chromatophore we carried out a statistical analysis using the program GeneMerge [63]. First, we classified each of the genes in both genomes (the chromatophore of *Paulinella chromatophora* CCAC 0185 [5] and *Synechococcus sp*. WH 5701) according to the functional categories of KEGG orthology (http://www.genome.jp/kegg/ko.html). Then we carried out the statistical analysis with GeneMerge. GeneMerge is a program written in Perl which allows the identification of overrepresented functions or categories in a sample by using a hypergeometric distribution [63].

### Metabolic reconstruction of the *i*CV498 and the *i*CV265 models

A draft metabolic model was initially reconstructed by identifying orthologous genes between *Synechococcus sp*. WH 5701 and the metabolic model of *Synechocystis sp*. PCC 6803 (*i*JN678) [26]. Because this draft metabolic network had many inconsistencies we performed a manual refinement. This consisted in reviewing literature and databases to fill gaps in the model. We followed recommendations of [64].

The metabolic network of the endosymbiont was reconstructed by identifying orthologs between the chromatophore and *Synechococcus sp*. WH 5701. *Synechococcus sp*. WH 5701 is the closest free-living relative of the chromatophore with a sequenced genome [5].

The metabolic capabilities of both organisms were tested with Flux Balance Analysis [65]. FBA is an optimization algorithm based on lineal programming provided in the Matlab COBRA toolbox [66]. FBA determines the flux distribution of all reactions in the model by maximizing an objective function [30].

The functionality of metabolic models is evaluated by their capacity to produce every metabolite that is necessary for in silico growth. For this, the biomass equation of *Synechocystis sp*. PCC 6803 was assigned as objective function in both models. In silico growth was simulated under autotrophic conditions with CO_2_ and photons uptake set to 3.7 mmol × gDW^−1^ × h^−1^ and 100 mmol × gDW^−1^ × h^−1^, respectively. These were restrictive metabolites in the systems. Nutrient assignment for metabolic functionality of the chromatophore was based on the literature [5] and metabolite requirements predicted by the model for in silico biomass production.

### Network robustness analysis

In both models, robustness to gene deletions was analyzed by using the function singleGeneDeletion of the COBRA toolbox. If deletion of a single gene decreases the biomass production over 99%, compared with wild type, this gene was consider as essential for biomass production.

### Simulation of metabolic reductive evolution in the chromatophore

To simulate genome reduction, we used the metabolic model of *Synechococcus sp*. WH 5701 (*i*CV498) as a proxy of the free-living ancestor of the chromatophore (Fig. 2). Genetic loss was simulated under Proposed Nutrients and Randomized Nutrients intracellular conditions. All nutrients were available simultaneously since the beginning of the simulations. The algorithm starts by randomly deleting a gene from the *i*CV498 model (i.e., setting its flux to zero) and then evaluating the impact of this deletion in the metabolic functionality by using FBA. If in silico growth rate in this network (lacking a gene) was equal to or above the growth rate of a free-living cyanobacteria (≥ 0.0884 h^−1^), then this gene was considered as non-essential and permanently removed. In contrast, if the growth rate was below 0.0884 h^−1^ then this gene was considered as essential and retained in the model. This process was repeated until each of the genes in the model was evaluated. The whole process is initiated 500 times which results in a population of 500 reduced metabolic networks.

Genetic concordance between the 500 reduced metabolic networks and chromatophore model (*i*CV265) was analyzed as in [30]. In each of the 500 simulations, a binary variable was assigned for each gene in *i*CV498 depending on whether the gene is predicted to be conserved or not among the 500 simulations. This allowed us to determine the number of occurrences that a gene is predicted as essential in the 500 simulated reduced networks.

Measures of sensitivity and specificity were obtained calculating the fraction of true-positives (fraction of genes predicted to be conserved by the simulations and present in *i*CV265) and false-positives (fraction of genes predicted by the simulations and not present in *i*CV265) for every cutoff (minimal fraction of simulated genomes in which a gene must be present to be predicted as conserved in *i*CV265). Figure 5 plots true-positive and false-positive (1-specificity) predictions for every cutoff (1 to 500) to form a ROC curve. The area under the curve represents how well the simulations recover gene content in *i*CV265. The area under the curve was empirically calculated as in [67].

### Simulation of metabolic integration of the chromatophore with its host

We performed this analysis by using the same algorithm used in the simulation of reductive evolution. However, this analysis was performed only in Proposed Nutrient conditions (Fig. 2). In addition, a reaction simulating hexose export from the chromatophore to the host was defined as objective function and the growth rate equation (biomass equation) was fixed to 0.0884 h^−1^. Also, in this simulation, a non-essential gene was defined as one whose deletion does not affect the growth rate (0.0884 h^−1^) and the hexose export. Specifically, the rate of hexose export could vary while being always greater than zero. Otherwise, the gene was defined as essential.

In this analysis the model does not have access to all 13 nutrients at the same time from the beginning of the simulation. Instead, we randomly allow the model to have access to one of the 13 Proposed Nutrients (Fig. 2) and subsequently applied our algorithm of reductive evolution. Once we evaluated the impact of singly deleting each one of the genes, we randomly allowed the model to have access to a second nutrient and newly applied our algorithm of reductive evolution. The analysis stops when *i*CV498 has access to all 13 nutrients and all genes have been tested for essentiality.

## Additional files

Additional file 1: Metabolic model of *Synechococcus sp*. WH 5701 (*i*CV498). (XLSX 187 kb)

Additional file 2: Metabolic model of the chromatophore (*i*CV265). (XLSX 121 kb)

Additional file 3: Figure S1 and S2 and Tables S1. (DOCX 149 kb)
