# Supplementary material for: Natural selection drove metabolic specialization of the chromatophore in Paulinella chromatophora
Source: BMC Evol Biol. 2017 Apr 14;17:99. doi: 10.1186/s12862-017-0947-6 (PMC5392233; doi:10.1186/s12862-017-0947-6)
Supplement: Supplementary file 3 — Figure S1 and S2 and Tables S1. (DOCX 149 kb) [file 12862_2017_947_MOESM3_ESM.docx]

Natural selection drove metabolic specialization of the

chromatophore in *Paulinella chromatophora*

Cecilio Valadez-Cano^1^, Roberto Olivares-Hernández^2^, Osbaldo Resendis-Antonio^3,4^, Alexander DeLuna^5^ and Luis Delaye^1^*

Addtional information 3.

Supplementary information.


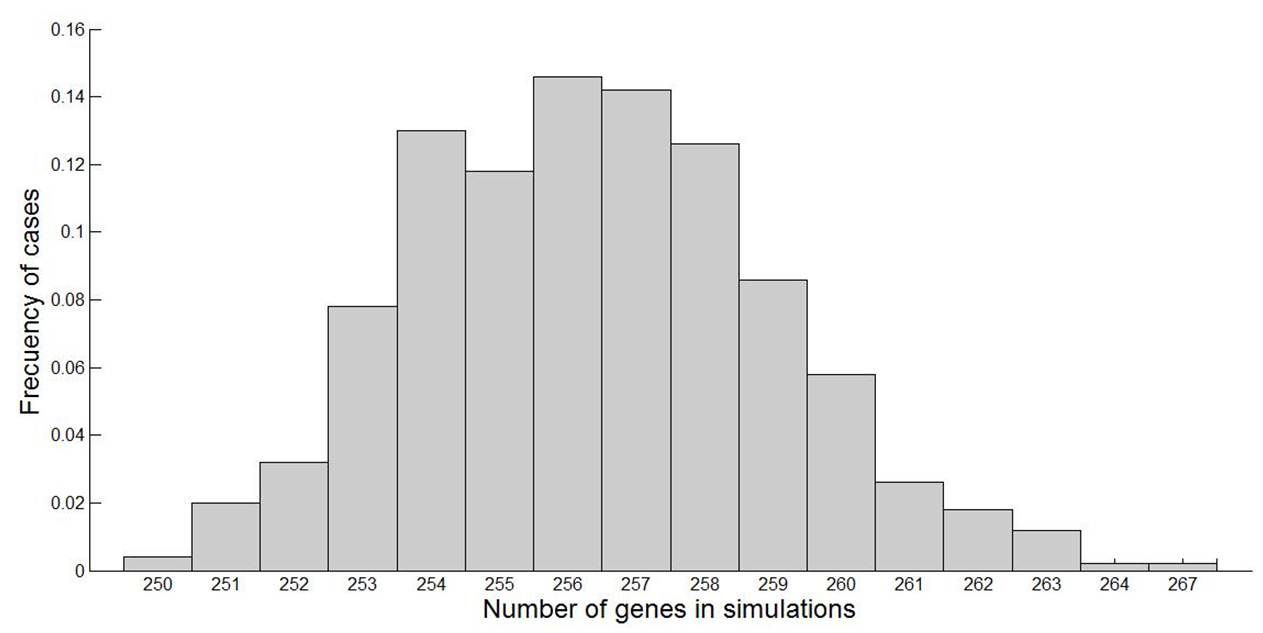


Figure S1. Distribution of genes in minimal networks. Minimal networks contain, in average 256.3 genes ± 2.63 (mean ± s.d.).


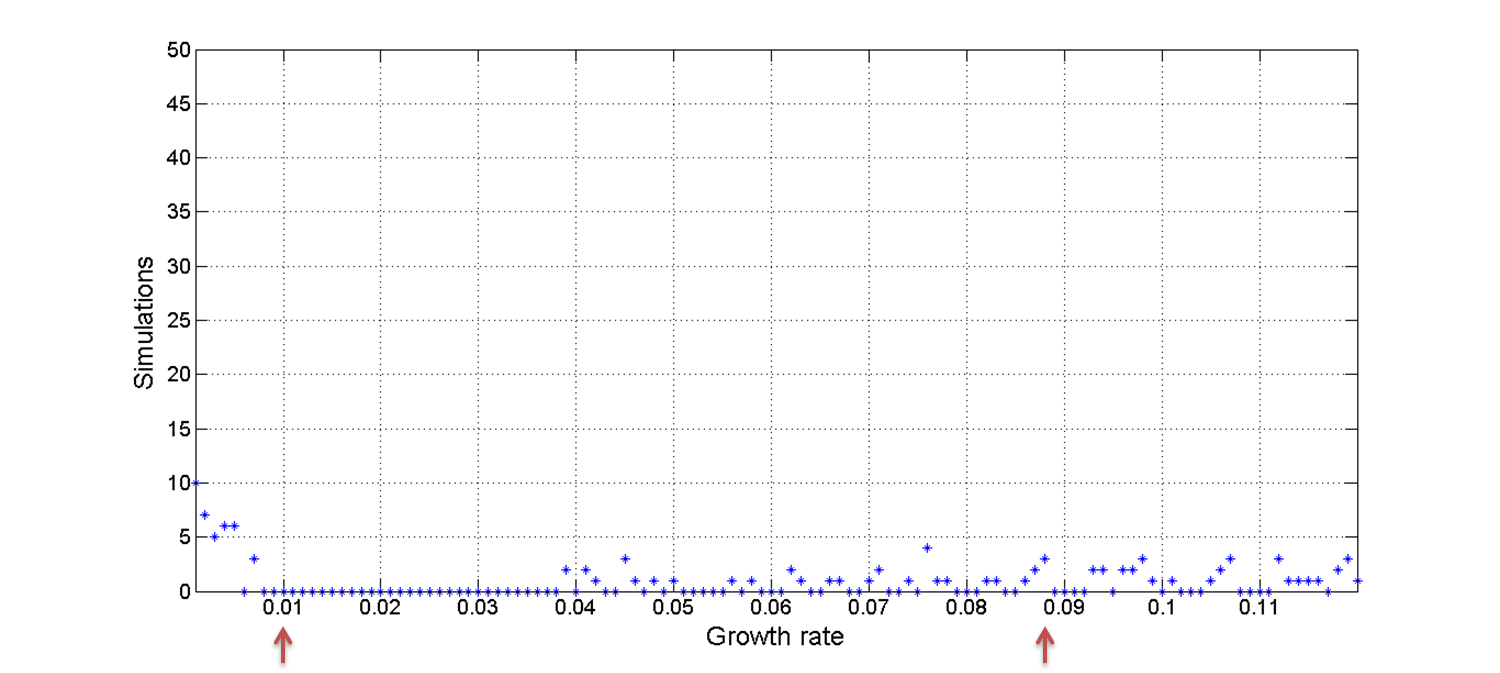


Figure S2. The arbitrary restriction of growth rate does not affect the number of simulations with hexose release capacity equal or higher than *i*CV265. Blue asterisks, number of simulations with hexose release capacity equal or higher than *i*CV265 in 50 simulations (Y axis) in different growth rate constraints (X axis). Red arrows represents growth rate of the algae *Chlorella sorokiniana* (≈ 0.01 h^-1^) and the cyanobacteria *Synechocystis sp.* PCC 6803 (≈ 0.088h^-1^)[1,2].

Table S1. Randomized Nutrients.

| Randomly assigned nutrients | |
| --- | --- |
| Lysine | UDP |
| Threonine | D-Galactose |
| Cysteine | 1,2-Diacyl-sn-glycerol (dioctadec-11-enoyl, n-C18:1) |
| Ornithine | O-Succinylbenzoyl-CoA |
| Valine | 2-Phosphoglycolate |
| 5-Phospho-alpha-D-ribose 1-diphosphate | 3-Oxohexadecanoyl-[acyl-carrier protein] |
| Zeaxanthin |  |

**Bibliography**

1. Kim S, Park J eun, Cho YB, Hwang SJ. Growth rate, organic carbon and nutrient removal rates of Chlorella sorokiniana in autotrophic, heterotrophic and mixotrophic conditions. Bioresour. Technol. [Internet]. Elsevier Ltd; 2013;144:8–13. Available from: http://dx.doi.org/10.1016/j.biortech.2013.06.068

2. Nogales J, Gudmundsson S, Knight EM, Palsson BO, Thiele I. Detailing the optimality of photosynthesis in cyanobacteria through systems biology analysis. Proc. Natl. Acad. Sci. U. S. A. [Internet]. 2012;109:2678–83. Available from: http://www.pnas.org/content/109/7/2678.abstract
